# Agreements between Industry and Academia on Publication Rights: A Retrospective Study of Protocols and Publications of Randomized Clinical Trials

**DOI:** 10.1371/journal.pmed.1002046

**Published:** 2016-06-28

**Authors:** Benjamin Kasenda, Erik von Elm, John J. You, Anette Blümle, Yuki Tomonaga, Ramon Saccilotto, Alain Amstutz, Theresa Bengough, Joerg J. Meerpohl, Mihaela Stegert, Kelechi K. Olu, Kari A. O. Tikkinen, Ignacio Neumann, Alonso Carrasco-Labra, Markus Faulhaber, Sohail M. Mulla, Dominik Mertz, Elie A. Akl, Dirk Bassler, Jason W. Busse, Ignacio Ferreira-González, Francois Lamontagne, Alain Nordmann, Viktoria Gloy, Heike Raatz, Lorenzo Moja, Shanil Ebrahim, Stefan Schandelmaier, Xin Sun, Per O. Vandvik, Bradley C. Johnston, Martin A. Walter, Bernard Burnand, Matthias Schwenkglenks, Lars G. Hemkens, Heiner C. Bucher, Gordon H. Guyatt, Matthias Briel

**Affiliations:** 1 Basel Institute for Clinical Epidemiology and Biostatistics, University Hospital of Basel, Basel, Switzerland; 2 Cochrane Switzerland, Institute of Social and Preventive Medicine (IUMSP), Lausanne University Hospital, Lausanne, Switzerland; 3 Department of Clinical Epidemiology and Biostatistics, McMaster University, Hamilton, Ontario, Canada; 4 Department of Medicine, McMaster University, Hamilton, Ontario, Canada; 5 Cochrane Germany, University of Freiburg, Freiburg, Germany; 6 Epidemiology, Biostatistics and Prevention Institute (EBPI), University of Zurich, Zurich, Switzerland; 7 Austrian Federal Institute for Health Care, Department of Health and Society, Vienna, Austria; 8 Departments of Urology and Public Health, Helsinki University Hospital and University of Helsinki, Helsinki, Finland; 9 Department of Internal Medicine, Pontificia Universidad Católica de Chile, Santiago, Chile; 10 Evidence-Based Dentistry Unit, Faculty of Dentistry, Universidad de Chile, Santiago, Chile; 11 Michael G. DeGroote Institute for Infectious Disease Research, McMaster University, Hamilton, Ontario, Canada; 12 Department of Internal Medicine, American University of Beirut, Beirut, Lebanon; 13 Department of Medicine, State University of New York at Buffalo, Buffalo, New York, United States of America; 14 Department of Neonatology, University Hospital of Zurich, Zurich, Switzerland; 15 Michael G. DeGroote Institute for Pain Research and Care, McMaster University, Hamilton, Ontario, Canada; 16 Department of Anesthesia, McMaster University, Hamilton, Ontario, Canada; 17 Epidemiology Unit, Department of Cardiology, Vall d’Hebron Hospital and Centro de Investigación Biomédica en Red de Epidemiología y Salud Publica (CIBERESP), Barcelona, Spain; 18 Centre de Recherche Clinique Étienne-Le Bel and Department of Medicine, Université de Sherbrooke, Sherbrooke, Quebec, Canada; 19 Institute of Nuclear Medicine, University Hospital of Bern, Bern, Switzerland; 20 IRCCS Orthopedic Institute Galeazzi, Milan, Italy; 21 Stanford Prevention Research Center, Stanford University, Stanford, California, United States of America; 22 Academy of Swiss Insurance Medicine, University Hospital of Basel, Basel, Switzerland; 23 Chinese Evidence-Based Medicine Center, West China Hospital, Sichuan University, Chengdu, China; 24 Department of Medicine, Innlandet Hospital Trust–Division Gjøvik, Oppland, Norway; 25 Institute of Health Policy, Management and Evaluation, University of Toronto, Toronto, Ontario, Canada; 26 Department of Anesthesia and Pain Medicine, Hospital for Sick Children, Toronto, Ontario, Canada; York University, CANADA

## Abstract

**Background:**

Little is known about publication agreements between industry and academic investigators in trial protocols and the consistency of these agreements with corresponding statements in publications. We aimed to investigate (i) the existence and types of publication agreements in trial protocols, (ii) the completeness and consistency of the reporting of these agreements in subsequent publications, and (iii) the frequency of co-authorship by industry employees.

**Methods and Findings:**

We used a retrospective cohort of randomized clinical trials (RCTs) based on archived protocols approved by six research ethics committees between 13 January 2000 and 25 November 2003. Only RCTs with industry involvement were eligible. We investigated the documentation of publication agreements in RCT protocols and statements in corresponding journal publications. Of 647 eligible RCT protocols, 456 (70.5%) mentioned an agreement regarding publication of results. Of these 456, 393 (86.2%) documented an industry partner’s right to disapprove or at least review proposed manuscripts; 39 (8.6%) agreements were without constraints of publication. The remaining 24 (5.3%) protocols referred to separate agreement documents not accessible to us. Of those 432 protocols with an accessible publication agreement, 268 (62.0%) trials were published. Most agreements documented in the protocol were not reported in the subsequent publication (197/268 [73.5%]). Of 71 agreements reported in publications, 52 (73.2%) were concordant with those documented in the protocol. In 14 of 37 (37.8%) publications in which statements suggested unrestricted publication rights, at least one co-author was an industry employee. In 25 protocol-publication pairs, author statements in publications suggested no constraints, but 18 corresponding protocols documented restricting agreements.

**Conclusions:**

Publication agreements constraining academic authors’ independence are common. Journal articles seldom report on publication agreements, and, if they do, statements can be discrepant with the trial protocol.

## Abstract 2

In a document analysis of trial protocols and publications, Erik von Elm and colleagues investigate the potential impact of publication agreements between industry sponsors and academic investigators.

## Author Summary

**Why Was This Study Done?:**

- Many randomized trials are designed and sponsored by for-profit companies that contract academic investigators to recruit and manage patients.
- Clinical research under these circumstances is a business transaction that bears the potential for conflicts of interest, in particular with respect to trial publication.
- Besides evidence from a small sample, it was unclear how often trial protocols included publication agreements between industry and academic investigators, whether these agreements constrained the investigators’ publication rights, and how consistent such agreements stated in trial protocols were with those reported in corresponding publications.

**What Did the Researchers Do and Find?:**

- We investigated publication agreements in 647 randomized trial protocols approved in 2000–2003 by six research ethics committees in Switzerland, Canada, and Germany, and in 388 corresponding journal publications.
- Seventy percent of protocols mentioned an agreement on publication rights between industry and academic investigators; in 86% of those agreements, industry retained the right to disapprove or at least review manuscripts before publication.
- Seventy-four percent of agreements documented in protocols were not mentioned in corresponding journal articles.

**What Do These Findings Mean?:**

- Publication agreements constraining academic investigators’ independence are incompletely reported in publications; this may compromise the scientific evidence base established by randomized clinical trials.
- More transparency on publication constraints is warranted.
- Half of the included journal articles were published before 2008, leaving open the possibility that these findings do not reflect current reporting practice.

## Introduction

Many randomized clinical trials (RCTs) are designed and sponsored by for-profit companies [1–3]. Companies typically contract academic investigators to identify, recruit, and manage patients. Clinical research under these circumstances is a business transaction that bears the potential for conflicts of interest, including those regarding the publication of trial results [4].

Academic investigators’ careers depend on publication of research results in peer-reviewed journals. For-profit companies aim for approval of new products by regulating agencies or expansion of product indications [5]. Publication of favourable results is also part of companies’ marketing strategy [6–8]. Industry-sponsored trials are less likely to be published than those not sponsored by industry [2,3], the likelihood of publication of outcome data can be related to the direction of the results [1,9,10], and discrepancies between trial reports submitted to regulatory agencies and journal publications occur [11].

To promote transparency in the arrangements between industry and academia, reporting guidelines recommend disclosure of potential conflicts of interest of authors and funders [12,13]. Complete reporting helps readers of journal articles judge how potential conflicts of interest may influence the reporting of trial results. Complete reporting includes details regarding both (i) agreements between industry and study investigators that may affect the publication of results and (ii) co-authorship of industry employees. Several high-impact journals insist that authors of trial reports disclose the sponsor’s role in the study [14–16]. The International Committee of Medical Journal Editors (ICMJE) recommends that investigators should avoid agreements with sponsors that interfere with full access to the dataset and the investigators’ ability to conduct analyses, interpret the results, and submit the manuscript for publication [17]. Further, it has been suggested that journal editors should review protocols or contracts with a focus on publication rights: “Editors may choose not to consider an article if a sponsor has asserted control over the authors’ right to publish” [18].

Previous studies have documented constraints on the publication rights of academic investigators in industry-sponsored RCTs [19,20]. Gøtzsche et al. investigated such constraints in 88 RCT protocols approved by two Danish research ethics committees—44 in 1994/1995 and 44 in 2004—and subsequent journal publications. They found that industry sponsors could have prevented publication in half of the trials. However, this study was restricted to a relatively small sample from a single country [21].

In this article, we consider agreements on publication rights in a cohort of RCT protocols approved by six research ethics committees (RECs) between 13 January 2000 and 25 November 2003 in three countries and the reporting of these agreements in corresponding publications. We also investigate co-authorship by industry employees and the concordance of statements regarding publication rights between trial protocols and corresponding publications.

## Methods

### Ethical Approval

The participating RECs approved the study or explicitly stated that no ethical approval was necessary.

### Aims

We aimed to investigate (i) the existence and types of publication agreements in trial protocols, (ii) the completeness and consistency of the reporting of these agreements in subsequent publications, and (iii) the frequency of co-authorship of industry employees.

### Study Design

Previous publications describe in detail the design of this retrospective cohort study [2,22]. In brief, we examined RCT protocols approved between 13 January 2000 and 25 November 2003 by six RECs in Switzerland (Basel, Lucerne, Zurich, and Lausanne), Germany (Freiburg), and Canada (Hamilton) (S1 Table). Of these RECs, all but one (Lucerne) were responsible for human research in large university centres and hospitals in their respective catchment areas. The REC in Lucerne covered an academic teaching hospital and other health research in Central Switzerland. We used our existing contacts to establish this convenience sample of RECs. We determined the completion status and publication history of RCTs as of 27 April 2013 by using information available in REC files and by conducting comprehensive searches for corresponding publications in databases (MEDLINE, Embase, the Cochrane Central Register of Controlled Trials, CINAHL, the Allied and Complementary Medicine Database, Google Scholar, and topic-specific databases) and trial registers (ClinicalTrials.gov and the WHO International Clinical Trials Registry Platform). Two independent investigators determined whether identified publications matched the corresponding protocol. In the case of unclear trial completion or publication status, the REC in charge contacted the investigators using a standardized questionnaire.

### Eligibility Criteria for Protocols and Subsequent Publications

In the present analysis, we considered only RCT protocols that clearly documented industry involvement in the design, support, or conduct of the trial (e.g., sponsorship, logistical support, partial funding, or supply of a drug/device). We excluded protocols of studies that (i) compared different doses or routes of administration of the same drug (such as early dose-finding studies), (ii) enrolled only healthy volunteers, (iii) were never started, or (iv) were still ongoing as of April 27, 2013. With respect to multiple corresponding publications, we included only the primary full publication that reported the results from the randomized comparison, and excluded research letters, letters to the editor, and conference abstracts. In the case of more than one full publication, we considered the first publication that included results for the RCT’s primary outcomes.

### Definitions

We defined documentation of publication rights as any statement about an agreement between an industry sponsor and the academic investigators regarding the publication of trial results. We classified eligible RCT protocols according to the sponsoring party (investigator or industry) that assumed formal responsibility for the conduct of the trial; the sponsoring party was identified from the following types of information in the protocol: a clearly named sponsor, a prominently displayed company or institution logo, the affiliations of protocol authors, statements about data ownership or publication rights, and statements about full funding by industry or public funding agencies. Disagreements were resolved by consensus or by involving a third investigator as arbitrator (B. K., M. B., or E. v. E.). Investigator-sponsored trials eligible for this study had at least some industry funding, provision of study drugs, or logistical support from industry; industry did not, however, entirely fund these trials.

### Information Collected about Publication Agreements

We recorded the presence or absence of any publication agreement between the academic investigators and industry documented in protocols and reported in publications. If reviewers identified such documentation, they assigned it to the most appropriate of the following four mutually exclusive categories: (i) The industry partner retains the right to disapprove any submission for publication (this included any publication using trial data [abstracts or manuscripts for journal publications]). (ii) The industry partner retains the right to at least review and comment on any manuscript/abstract before publication (further constraints may have been possible, but were not clear from the source). (iii) No constraints by the industry partner; in particular, no right to withhold the submission from publication. (iv) Reference to a separate publication agreement document between the industry partner and the investigator, with no further details. Prompted by a reviewer’s comment and considering the considerable ambiguity of original statements, we collapsed categories (i) and (ii) in the analyses herein and labelled the combined category as follows: the industry partner retains the right to disapprove or at least review any abstract or manuscript for publication. Examples of publication agreements in trial protocols are provided in S2 Table.

### Data Extraction Process and Search for Publications

Twelve investigators trained in clinical research methodology extracted data from the included trial protocols and correspondence between the RECs and local investigators at the respective centres. To increase consistency in data extraction, pairs of two reviewers extracted the initial 30% of data independently and compared results to achieve consensus; disagreements were resolved by discussion and consultation with an arbitrator (B. K., M. B., or E. v. E.) if necessary. If the available REC files provided no information about the publication status of a trial, we conducted comprehensive searches of electronic databases and surveyed investigators to find any corresponding publications as of April 27, 2013. Twenty-two investigators trained in clinical research methodology extracted data from all corresponding publications, independently and in duplicate; disagreements were resolved by discussion to achieve consensus or by consultation with an arbitrator (B. K., M. B., or E. v. E.). None of the reviewers among the teams extracted data from both a protocol and its corresponding publication.

### Statistical Analysis

We summarized binary data as frequencies and proportions and continuous data as medians and interquartile ranges. Publication agreements are described for protocols and corresponding publications separately. To explore differences regarding publication agreements, we stratified by industry versus investigator sponsorship. Prompted by reviewer comments, we additionally explored the differences regarding publication agreements stratified by the extent of industry funding, categorized as (i) provision of medication/device only, (ii) partially funded, beyond medication/device but not whole trial, and (iii) full funding of trial. When analyzing concordance between protocols and publications, we considered only those protocols in which the publication agreement was accessible to us. We used the statistical programme R version 3.1.2 (https://www.r-project.org/) for all analyses. Data are deposited in the Dryad Digital Repository: doi:10.5061/dryad.3s6j7 [23].

## Results

### Trial Characteristics and Publications

**Fig 1:**
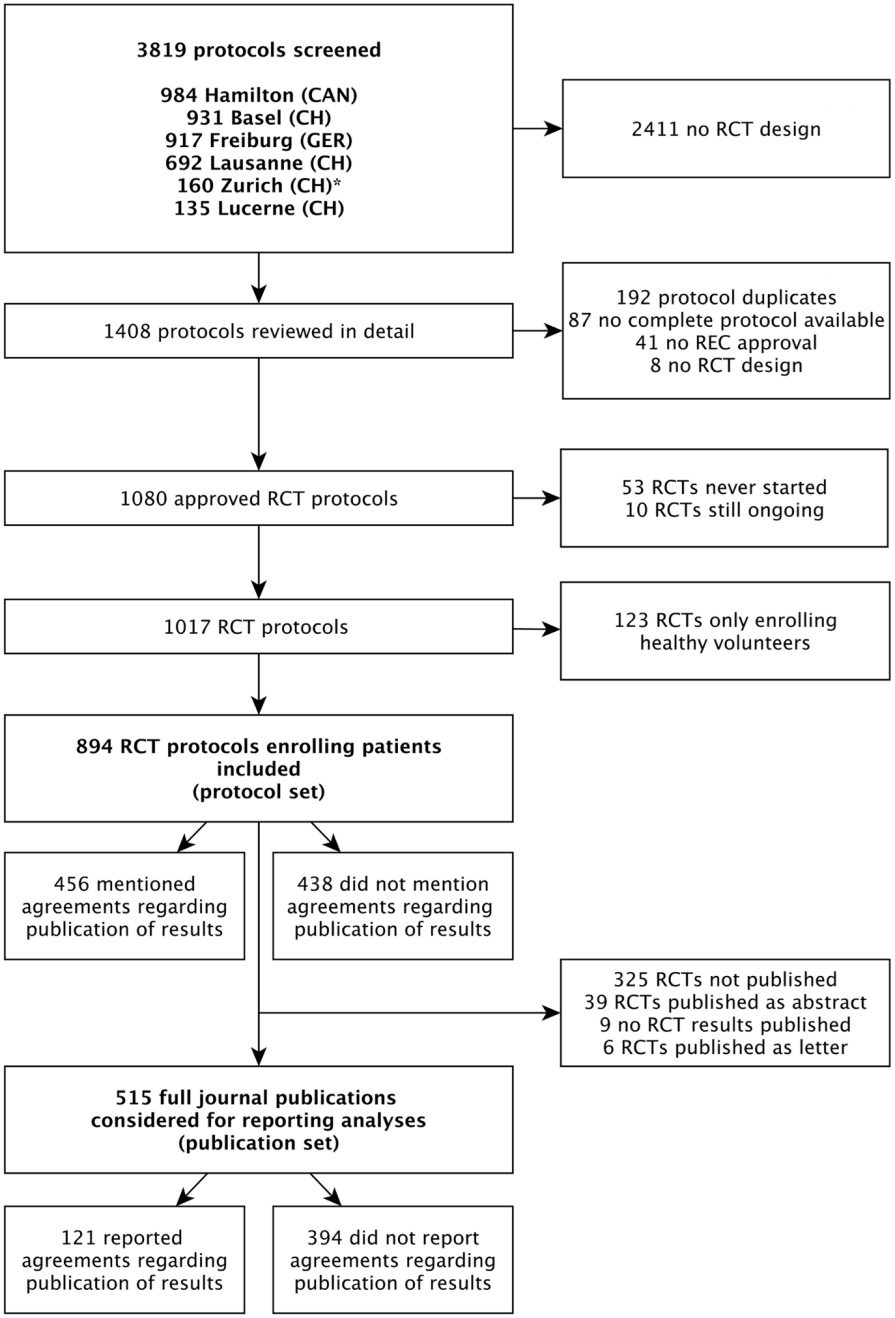
Study flow of RCT protocols and publications. For the Zurich REC, we included RCT protocols only from the two subsidiary RECs responsible for paediatric and surgical RCTs.

Of 894 RCT protocols involving patients approved by the six RECs between 13 January 2000 and 25 November 2003, 247 (27.6%) had no industry involvement and were excluded. We included 647 (72.4%) protocols, of which 456 (70.5%) mentioned publication agreements (Fig 1). RCTs with protocols that mentioned agreements were on average larger (median sample size 360 versus 222) and more often multicentre trials (439/456 [96.3%] versus 150/191 [78.5%]). Most RCT protocols that mentioned an agreement (417/456 [91.4%]) were industry sponsored (Table 1). In all 39 investigator-sponsored trial protocols that mentioned an agreement, one or more industry partners provided drugs or logistical support. For the included 647 RCTs, we found 388 (60.0%) full journal articles (328 [60.0%] for 547 industry-sponsored RCTs; 60 [60%] for 100 investigator-sponsored RCTs).

**Table 1:** Characteristics of included randomized clinical trials as extracted from trial protocols.

Characteristic	No Documented Publication Policy (*n =* 191)	Documented Publication Policy (*n =* 456)	Total (*n =* 647)
**Trial sample size**			
Median (interquartile range)	221.5 (100, 600)	360 (160, 712.5)	318 (125, 675)
**Centre status**			
Multicentre trial	150 (78.5%)	439 (96.3%)	589 (91.0%)
Single-centre trial	40 (20.9%)	16 (3.5%)	56 (8.7%)
Unclear	1 (0.5%)	1 (0.2%)	2 (0.3%)
**Study type**			
Superiority trial	133 (69.6%)	334 (73.2%)	467 (72.2%)
Non-inferiority/equivalence trial	30 (15.7%)	89 (19.5%)	119 (18.4%)
Unclear	28 (14.7%)	33 (7.2%)	61 (9.4%)
**Sponsorship**			
Industry	130 (68.1%)	417 (91.4%)	547 (84.5%)
Investigator	61 (31.9%)	39 (8.6%)	100 (15.5%)
**Extent of industry funding**			
Fully industry funded	125 (65.4%)	401 (87.9%)	526 (81.3%)
Partially industry funded, beyond medication/device	22 (11.5%)	22 (4.8%)	44 (6.8%)
Only medication/device funded	23 (12.0%)	21 (4.6%)	44 (6.8%)
Unclear	21 (11.0%)	12 (2.6%)	33 (5.1%)
**REC**			
Freiburg	61 (31.9%)	134 (29.4%)	195 (30.1%)
Basel	40 (20.9%)	144 (31.6%)	184 (28.4%)
Hamilton	48 (25.1%)	68 (14.9%)	116 (17.9%)
Lausanne	30 (15.7%)	76 (16.7%)	106 (16.4%)
Lucerne	7 (3.7%)	17 (3.7%)	24 (3.7%)
Zurich	5 (2.6%)	17 (3.7%)	22 (3.4%)
**General medical field**			
Medical (adult)	151 (79.1%)	391 (85.7%)	542 (83.8%)
Surgical (adult)	11 (5.8%)	30 (6.6%)	41 (6.3%)
Paediatrics	29 (15.2%)	35 (7.7%)	64 (9.9%)
**Five most common specific medical fields** *			
Oncology	20 (10.5%)	84 (18.4%)	104 (16.1%)
Cardiovascular	29 (15.2%)	55 (12.1%)	84 (13.0%)
Infectious disease	21 (11.0%)	53 (11.6%)	74 (11.4%)
Neurology	13 (6.8%)	42 (9.2%)	55 (8.5%)
Endocrinology	9 (4.7%)	40 (8.8%)	49 (7.6%)

Data are presented as frequencies (column percentages) unless stated otherwise.

*A comprehensive list of all medical fields is provided in S1 Fig.

### Types of Agreements Mentioned in Protocols and Publications

In 393 of 456 (86.2%) protocols, the industry partner had the right to disapprove or at least to review publications proposed by academic investigators (Table 2). Publication agreements without any constraints by the industry partner on the academic investigator were documented in 39 (8.6%) RCT protocols (14/417 [3.4%] industry-sponsored RCTs; 25/39 [64.1%] investigator-sponsored RCTs). Twenty-four (5.3%) protocols mentioned separate agreement documents that were not accessible to us.

**Table 2:** Types of publication agreements and industry employee co-authorship as documented in trial protocols and reported in journal publications.

Publication Agreement or Industry Co-authorship	Protocols			Publications		
	417 Industry Sponsored	39 Investigator Sponsored	456 Total	78 Industry Sponsored	20 Investigator Sponsored	98 Total
Industry has the right to disapprove or at least review any publication	383 (91.8%)	10 (25.6%)	393 (86.2%)	58 (74.4%)	3 (15.0%)	61 (62.2%)
No publication constraints by industry	14 (3.4%)	25 (64.1%)	39 (8.6%)	20 (25.6%)	17 (85.0%)	37 (37.8%)
Separate agreement mentioned in protocol	20 (4.8%)	4 (10.3%)	24 (5.3%)	Not applicable	Not applicable	Not applicable
At least one industry employee as co-author of publication	Not applicable	Not applicable	Not applicable	65 (83.3%)	4 (20.0%)	69 (70.4%)

Data are presented as frequencies (column percentages).

We only included protocols and publications that documented/reported agreements between industry and academic investigators on publication policies; therefore, 191 protocols (130 industry-sponsored RCTs; 61 investigator-sponsored RCTs) and 290 publications (250 industry-sponsored RCTs; 40 investigator-sponsored RCTs) were excluded.

Of 388 full journal publications, 98 (25.3%) mentioned an agreement regarding the publication of trial results and 290 (74.7%) did not. For 61 of the 98 (62.2%) trials that mentioned an agreement, authors reported that the industry partner had the right to disapprove or at least to review any publication. In 37 (37.8%) publications, the author statement suggested unrestricted publication rights (20/78 [25.6%] industry-sponsored RCTs; 17/20 [85.0%] investigator-sponsored RCTs) (Table 2). The distribution of RCTs with different types of publication agreements by extent of industry funding is displayed in S3 Table.

In 260 of 388 (67.0%) publications, at least one co-author was an industry employee (253/328 [77.1%] industry-sponsored trials, 7/60 [11.7%] investigator-sponsored trials). The median proportion of industry employees among all authors for journal publications was 25% (interquartile range, 17% to 40%); it was 30% for industry-sponsored RCTs and 10% for investigator-sponsored RCTs. In 14 of the 37 (37.8%) publications in which there was a statement that suggested unrestricted publication rights, at least one co-author was an industry employee.

### Concordance between Protocols and Publications

Table 3 displays the concordance of information about publication agreements between protocols and subsequent journal publications. For this analysis, we excluded 191 protocols that did not mention a publication agreement and 24 protocols that mentioned a separate publication agreement document that was not accessible to us. This resulted in 432 protocols; of these, 268 had a corresponding journal publication. In 240 of these 268 (89.6%) protocols, the industry partner had the right to disapprove or at least review publications (Table 3). In most cases (177 of 240 [73.8%]), this agreement was not mentioned in the subsequent publication. Of all 71 journal articles that mentioned a publication agreement, 52 (73.2%) included a statement that was in concordance with the documentation in the protocol. In 25 publications, the author statement suggested no constraints, but for 18 of these publications, the corresponding protocol documented a restricting agreement (see S4 Table for excerpts from protocols and publications). In 28 (10.4%) of 268 protocols, the protocol documented that academic investigators were free of any constraints. This was reflected in the corresponding publications in seven instances (25.0%). In six of 268 publications (2.2%), the authors’ statement of unrestricted publication rights was in concordance with what was documented in the protocol and no industry employee was listed as a co-author; five of these were investigator-sponsored RCTs.

**Table 3:** Protocols with publication agreements and the reporting of these agreements in subsequent journal publications.

Documented in Protocol	Reported in Publication			Total Published*	Total with No Publication Found*	All
	Industry Had the Right to Disapprove or at Least Review Any Publication	No Publication Constraints by Industry	Agreement Not Reported			
Industry had the right to disapprove or at least review any publication	45 (18.8%)^$^	18 (7.5%)	177 (73.8%)	240 (61.1%)	153 (38.9%)	393
No publication constraints by industry	1 (3.6%)	7 (25.0%)^$^	20 (71.4%)	28 (71.8%)	11 (28.2%)	39
Total	46	25	197	268	164	432

We excluded 215 protocols that referred to a separate agreement document or did not mention anything about publication agreements. Data are presented as frequencies (row percentages, with column “Total Published” in the denominator) unless stated otherwise.

*Row percentages based on denominator in column “All”.

^$^Concordant statements in protocol and publication.

## Discussion

### Summary of Findings

Of 647 RCT protocols that were approved between 13 January 2000 and 25 November 2003 by six RECs in three countries and sponsored or supported by industry, 86% documented publication constraints reserving the right of the industry partner to review the manuscript or allowing the industry partner to disapprove the manuscript. Most agreements (74%) documented in protocols remained unreported in subsequent publications. In 18 instances, author statements in publications suggested no constraints by industry partners while protocols actually documented such constraints. Moreover, at least one co-author was an industry employee in two-thirds of journal publications and in one-third of publications in which statements suggested no publication constraints for academic authors. This suggests that, irrespective of the agreement in the protocol, the industry partner could influence publication content and submission decisions through co-authorship.

### Strengths and Limitations

Our data were collected as part of a large international cohort involving six RECs that allowed full access to trial protocols and filed correspondence between the local academic investigator and the REC in charge [2,22]; only investigators’ brochures were exempt from assessment. Because unrestricted access to trial protocols is necessary to maintain scientific rigor [24], we did not ask trialists and sponsors for permission to access their protocols. Doing so could have introduced bias because those with poor reporting practices may not have allowed additional scrutiny. Further strengths of our study include the use of trained methodologists for data collection and independent and duplicate data extraction from identified publications. Finally, our sample included RCTs from various fields of clinical medicine, thus enhancing the generalizability of our results.

Our study has limitations: First, separate agreement documents or legal contracts specifying publication rights were not available to us. However, only 5% of protocols that mentioned publication agreements referred to such separate documents. To the extent that protocols failed to refer to separate documents, we may have underestimated the overall prevalence of publication agreements. Second, we did not approach the local investigators submitting protocols to the REC to inquire whether their publication rights were actually constrained by the industry partner. Therefore, we cannot estimate to what extent the documented agreements impacted the content of publications or led to delays in publication or to non-publication of trial results. Third, the wording of the agreements in protocols was very heterogeneous. We extracted the original text only to provide examples but used our judgement to classify the statements according to four prespecified categories. Because of the considerable ambiguity of the original statements, we eventually collapsed two categories into one. Fourth, we used a convenience sample of six RECs that were—to our knowledge—not in any way particular. Five of them had authority for large university hospitals in their catchment areas, and one was responsible for a large teaching hospital. We cannot say whether these RECs are representative of other RECs in their own or other countries. Fifth, all included protocols were approved from 13 January 2000 and 25 November 2003, and half of the included publications were from the year 2007 or before, leaving the possibility that a substantial proportion of our sample no longer reflects current practices of reporting of publication agreements. In particular, the increasing demand for transparency in the reporting of clinical trials through guidelines and journal policies may have positively influenced current practice. Sixth, exploring the influence of industry co-authors in the writing process would require additional qualitative research, for example, interviews with author teams; however, this was not part of the present study. Seventh, we did not collect information regarding associated data-sharing agreements. The nature of such agreements might have influenced the interpretation of the publication agreements that were the focus of our study. Finally, we previously published the protocol of the overall project but not details about the present sub-study [22]. Therefore, we kept our analysis descriptive while including our data extraction forms detailing all collected variables in S5 Table for interested readers.

### Comparison with Other Studies

Gøtzsche et al. reported that 91% of 44 protocols from industry-sponsored trials approved by two RECs in 1994/1995 included constraints on publication rights, but none of the associated publications reported these constraints [21]. In our larger and more recent sample, authors provided statements about agreements on publication rights in about a quarter of journal publications.

Based on survey data from 108 US medical schools, Schulman et al. reported that academic institutions routinely engage in industry-sponsored research that fails to adhere to ICMJE guidelines regarding trial design, access to data, and publication rights [20]. In another survey focusing on institutional policies, Mello et al. approached 122 US medical schools in 2004 [19]. Of the 107 schools that participated, approximately 85% stated that they would not approve contractual provisions giving industry sponsors the authority to revise manuscripts or decide whether results should be published [19]. In the remaining 15%, however, the responsible office would allow such constraints. In our sample, 86% of industry-sponsored trial protocols documented that the sponsor retained the right to disapprove or at least review any resulting publication. The key role of academic medical centres in maintaining scientific integrity has been outlined in a policy proposal regarding their partnership with industry sponsors [25]. This proposal does not, however, explicitly address the issue of publication rights.

### Implications

Previous publications have documented misleading presentations of evidence, sometimes referred to as “spin” [26]. In industry-supported trials, spin can be due to conflicts of interest resulting from the funding arrangements [27]. Publication restrictions represent another form of conflict of interest, as reflected in ICMJE’s recent recommendation: “Authors should avoid entering in to agreements with study sponsors, both for-profit and non-profit, that interfere with authors’ access to all of the study’s data or that interfere with their ability to analyze and interpret the data and to prepare and publish manuscripts independently when and where they choose” [17]. Publication agreements may contribute to the presentation of results in misleading ways that favour the interests of a sponsor [28]. This is clearly a possibility when a trial’s industry sponsor has the right of disapproval, which restricts academic freedom in general.

Industry funding disclosed in trial manuscripts may represent a red flag for editors who are to decide about acceptance. If publication restrictions are acknowledged, this may be another. In turn, omission of such statements—or the presence of content that contradicts existing publication restrictions—deprives clinicians, guideline developers, and policy makers of such an alert, and may be deliberate.

We acknowledge that there are instances, in which restricting the investigators’ right to publish is appropriate, e.g., separate publication of subsets of data from a multicentre trial could be confusing. Such restrictions should, however, be limited in time, e.g., until completion of the trial, including publication of its main results. Pending patent applications may be another justification for a time-limited delay of publication, but can certainly not excuse withholding it completely. We suggest that a general reference to confidentiality in the protocol without further explanation is not sufficient to justify restriction or delay of publication.

Besides the issues of publication rights, co-authorship by industry employees on published trial reports allows the industry editorial influence. This issue also needs consideration in the context of possible reporting bias—in the light of our findings, journal editors should be aware of this issue.

The cooperation between industry and academic investigators can be very fruitful and can lead to great improvements in medical care. RECs have a crucial role in ensuring the ethical conduct of clinical research and should therefore also consider whether commercial sponsors’ rights to disapprove publication of trial results hamper the scientific process and erode trust in clinical research. Additionally, mandatory documentation of publication agreements in protocols (as proposed recently [29]) and trial registration could improve transparency. Further studies investigating publication agreements between academia and industry should also consider contracts in addition to approved protocols.

### Conclusions

Publication agreements are common in protocols of industry-sponsored RCTs. Journal publications of RCTs rarely provide readers with information about existing agreements, and when they do, statements can be discrepant with information in the corresponding trial protocols. Publication agreements constraining academic authors’ independence, and the incomplete reporting of such agreements in publications, may corrupt the scientific evidence base established by RCTs.

## Supporting Information

S1 Fig A comprehensive list of all medical fields. (TIFF)

S1 Table Participating research ethics committees. At the time of our study, the RECs in Basel and Lucerne were independent, but they have since been merged.
(DOCX)

S2 Table Examples of types of agreements on publication rights from trial protocols. (DOCX)

S3 Table Types of publication agreements and co-authorship of industry employees stratified by extent of industry funding. (DOCX)

S4 Table Comparison of original statements in trial protocols with those in corresponding publications in which the statement suggested an absence of publication constraints. (DOCX)

S5 Table Codebook of variables. Variables collected from protocols and publications for the entire cohort.
(PDF)

## Abbreviations

ICMJE: International Committee of Medical Journal Editors
RCT: randomized clinical trial
REC: research ethics committee
