# Supplementary figures and images for: Agreements between Industry and Academia on Publication Rights: A Retrospective Study of Protocols and Publications of Randomized Clinical Trials
Source: PLoS Med. 2016 Jun 28;13(6):e1002046. doi: 10.1371/journal.pmed.1002046 (PMC4924795; doi:10.1371/journal.pmed.1002046)

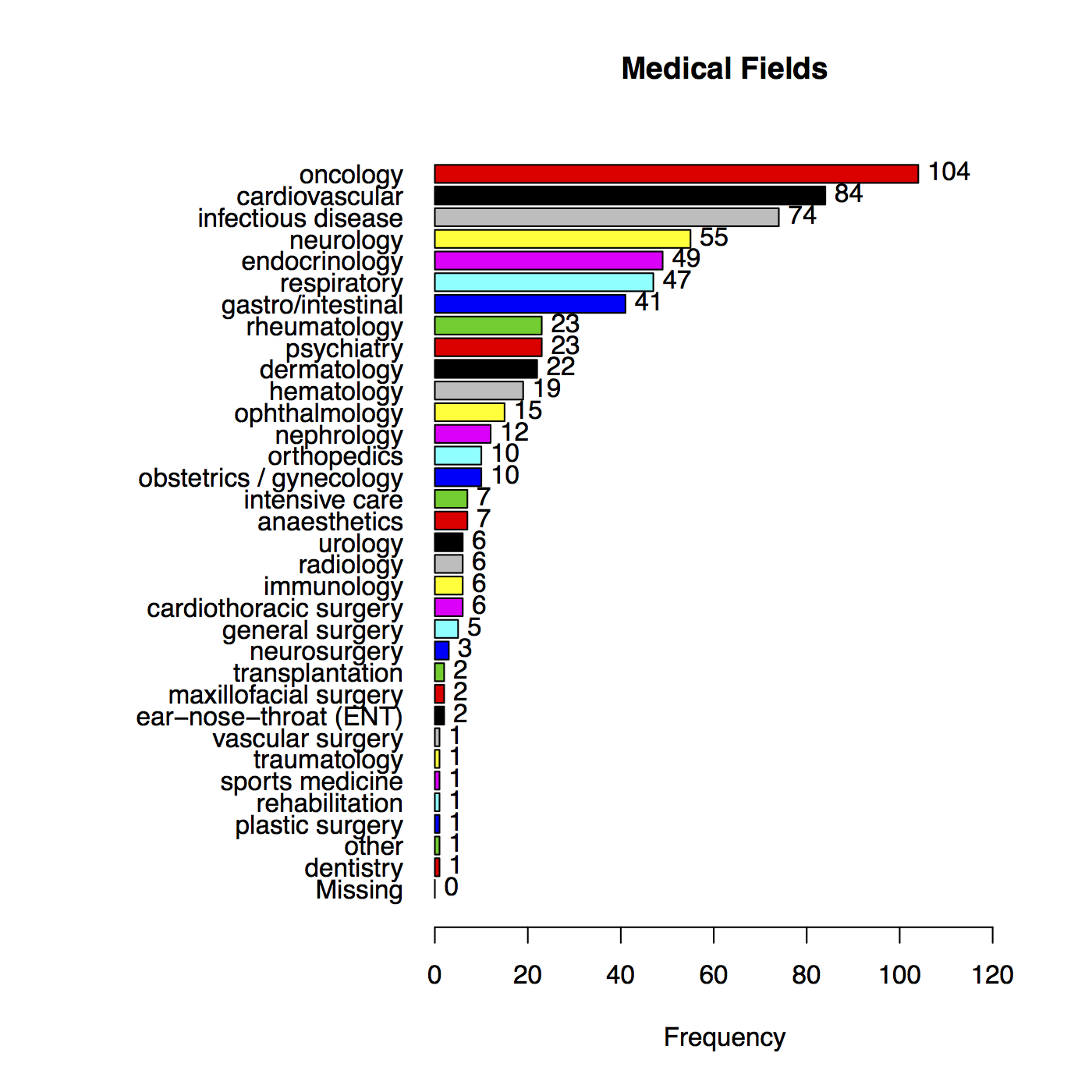

Supplement: S1 Fig — (TIFF) [file pmed.1002046.s001.tiff]
