# Supplementary material for: Agreements between Industry and Academia on Publication Rights: A Retrospective Study of Protocols and Publications of Randomized Clinical Trials
Source: PLoS Med. 2016 Jun 28;13(6):e1002046. doi: 10.1371/journal.pmed.1002046 (PMC4924795; doi:10.1371/journal.pmed.1002046)
Supplement: S1 Table — At the time of our study, the RECs in Basel and Lucerne were independent, but they have since been merged. (DOCX) [file pmed.1002046.s002.docx]

**S1 Table:** Participating research ethics committees. At the time of our study, the research Ethics committees in Basel and Lucerne were independent, but have meanwhile been merged.

| **Centre** | **Country** | **Responsible ethics committee** | **Current website** |
| --- | --- | --- | --- |
| Basel | Switzerland | Ethics committee for north-west and central Switzerland | http://eknz.ch/ |
| Zurich | Switzerland | Cantonal ethics committee Zurich | http://www.kek.zh.ch |
| Lausanne | Switzerland | Cantonal research ethics committee for human studies | http://www.cer-vd.ch/ |
| Lucerne | Switzerland | Ethics committee for north-west and central Switzerland | http://eknz.ch/ |
| Freiburg | Germany | Ethics committee university medical centre Freiburg | https://www.uniklinik-freiburg.de/ethikkommission/kontakt.html |
| Hamilton | Canada | Hamilton Integrated Research Ethics Board | http://www.hireb.ca/ |
