# Supplementary material for: Agreements between Industry and Academia on Publication Rights: A Retrospective Study of Protocols and Publications of Randomized Clinical Trials
Source: PLoS Med. 2016 Jun 28;13(6):e1002046. doi: 10.1371/journal.pmed.1002046 (PMC4924795; doi:10.1371/journal.pmed.1002046)
Supplement: S2 Table — (DOCX) [file pmed.1002046.s003.docx]

**S2 TABLE:** Examples of types of agreements on publication rights from trial protocols. The actual name of the industry partner has been replaced by “The Sponsor”.

| **Type of agreement** | **Example statements from protocols** |
| --- | --- |
| The industry partner retains the right to disapprove or at least review any abstract or manuscript for publication | 1. Trial 1: It is the policy of the Sponsor to encourage the presentation and/or publication of the results of their studies, using only clean, checked and validated data in order to ensure the accuracy of the results. At least forty-five (45) days in advance of proposed submission, the Investigator should forward a copy of the manuscript or abstract for review by the Sponsor, and, if necessary, delay publication or communication for a limited time in order to protect the confidentiality or proprietary nature of any information contained therein. The Sponsor may also request that the Sponsor's name and/or names of one or several of its employees appear or not appear in such publication. All study participants (Investigators and Committee members) give full authority to the sponsor for primary presentation and/or primary publication of results. No other publication is allowed before the primary publication. Any subsequent presentation or publication by a study participant must be approved by the sponsor and make reference to the study and the primary publication. 2. Trial 2: The lead investigator may publish/present the results of scientific investigations involving this clinical study, provided that confidential information of the Sponsor is not disclosed and that the Sponsor is provided with a copy of the manuscript sixty (60) days prior to presentation or submission for publication for review and approval, to insure that confidential information is not disclosed. 3. Trial 3: The Sponsor recognizes the importance of communicating medical trial data and, therefore, encourages their publication in reputable scientific journals and at seminars and conferences or congresses. Any results of medical trials with the sponsor's products and/or publication, lecture, manuscripts thereon shall be discussed by the investigator and the sponsor's representative(s) prior to publication. Regard shall be given to the sponsor's legitimate interests, e.g. containing optimal patent protection coordination of submissions to health authorities or with other ongoing trials in the same therapeutic field, protection of confidential data, and information, etc. The sponsor's comments shall be given without undue delay. If there is no consensus, the senior author of the manuscript and the sponsor's representative(s) shall further discuss and mutually agree on the final wording and/or disposition of the publication. The above procedure also applies to information on prematurely discontinued and other non-completed trials. Results from investigations shall not be made available to any third parties by the investigating team outside the publication procedure as set out above. The sponsor will not quote from publications by investigators in scientific information and/or promotional material without full acknowledgement of the source (i.e. author and reference). 4. Trial 4: Any formal presentation or publication of data collected from this trial will be considered as a joint publication by the investigator(s) and the appropriate personnel of the Sponsor. Authorship will be determined according to the number of eligible patients enrolled at each participating site. For multicenter studies, it is mandatory that the first publication is based on data from all centers, analyzed as stipulated in the protocol by the Sponsor’s statisticians, and not by the investigators themselves. Investigators participating in multicenter studies agree not to present data gathered from one center or a small group of centers before the full, initial publication, unless formally agreed to by all other investigators and the Sponsor. The Sponsor must receive copies of any intended communication in advance of publication (at least 15 working days for an abstract or oral presentation and 45 working days for a journal submission). The Sponsor will review the communications for accuracy (thus avoiding potential discrepancies with submissions to health authorities), verify that confidential information is not being inadvertently divulged and to provide any relevant supplementary information. Authorship of communications arising from pooled data will include members of each of the contributing centers as well as the Sponsor’s personnel. |
| No constraints by the industry partner; in particular, no right to withhold the submission for publication | 1. Trial 5: The principal investigator has the right to publish study data as first author. First authorship can be conceded by the principal investigator to one of the responsible investigators of the three study sites with the highest number of recruited patients into the trial. The top ten centres with regard to patient recruitment into the trial will have at least one co-author on the publication. 2. Trial 6: The results of this study will be submitted for publication in a major clinical journal after approval by the Steering Committee. Authors list of this paper will include the members of the Steering Committee, study coordination and at least one author from each participating center including more than 5 patients into this trial. From each center including more than 15 patients into the trial a second author will join the list. All other publications based on data from this clinical study are coordinated by the Steering Committee and the study coordination. To avoid any inconvenience between investigators all planned analyses or publications (including abstracts) have to be announced to the study coordination in advance. [Note by authors: Steering committee was a group of academic investigators.] |
| Reference to a separate publication agreement document between industry partner and investigator with no further details | 1. Trial 7: The conditions regulating dissemination of the information derived from this clinical study are described in the Clinical Trial Agreement. 2. Trial 8: Insurance coverage will be handled according to local requirements. Finance insurance and publication rights are addressed in separate agreements as appropriate. |
