# Supplementary material for: Agreements between Industry and Academia on Publication Rights: A Retrospective Study of Protocols and Publications of Randomized Clinical Trials
Source: PLoS Med. 2016 Jun 28;13(6):e1002046. doi: 10.1371/journal.pmed.1002046 (PMC4924795; doi:10.1371/journal.pmed.1002046)
Supplement: S3 Table — (DOCX) [file pmed.1002046.s004.docx]

**S3 TABLE:** Types of publication agreements and co-authorship of industry employees stratified by type of industry involvement

| **Agreements about publication policy in protocol or publication** | **Protocols** | | | | | **Publications** | | | | |
| --- | --- | --- | --- | --- | --- | --- | --- | --- | --- | --- |
|  | **Fully industry funded (total: 401)**  **N (%)** | **Medication/device only funded (total 21),**  **N (%)** | **With partial funding, beyond medication/device (total: 22),**  **N (%)** | **Unclear (total: 12),**  **N (%)** | **Total 456,**  **N (%)** | **Fully industry funded (total: 76),**  **N (%)** | **Medication/device only funded (total: 8),**  **N (%)** | **With partial funding, beyond medication/device (total: 8),**  **N (%)** | **Unclear (total: 6),**  **N (%)** | **Total 98,**  **N (%)** |
| Industry had the right to disapprove or at least to review any publication | 371 (92.5) | 10 (47.6) | 7 (31.8) | 5 (41.7) | 393 (86.2) | 57 (75.0) | 0 (0) | 1 (12.5) | 3 (50.0) | 61 (62.2) |
| No publication constraints by industry | 11 (2.7) | 10 (47.6) | 13 (59.1) | 5 (41.7) | 39 (8.6) | 19 (25) | 8 (100) | 7 (87.5) | 3 (50) | 37 (37.8) |
| Separate agreement mentioned in protocol | 19 (4.7) | 1 (4.8) | 2 (9.1) | 2 (16.7) | 24 (5.3) | Not applicable | Not applicable | Not applicable | Not applicable | Not applicable |
| At least one industry employee as co-author of publication | Not applicable | Not applicable | Not applicable | Not applicable | Not applicable | 62 (81.6) | 0 (0) | 1 (12.5) | 4 (66.7) | 67 (68.4) |

We only included protocols and publications that documented/reported agreements on publication policies; therefore 191 protocols (130 industry sponsored, 61 investigator sponsored) and 290 publications (250 industry sponsored, 40 investigator sponsored) were excluded. Numbers are frequencies (column percentages adding up to 100% excluding last row).
