# Supplementary material for: Agreements between Industry and Academia on Publication Rights: A Retrospective Study of Protocols and Publications of Randomized Clinical Trials
Source: PLoS Med. 2016 Jun 28;13(6):e1002046. doi: 10.1371/journal.pmed.1002046 (PMC4924795; doi:10.1371/journal.pmed.1002046)
Supplement: S4 Table — (DOCX) [file pmed.1002046.s005.docx]

**S4 TABLE:** Comparison of original statements in trial protocols with those in corresponding publications suggesting absence of publication constraints. The actual name of the industry sponsor has been replaced by “The sponsor”.

| **Excerpts from trial protocols** | **Excerpts from corresponding publication** |
| --- | --- |
| Should the investigator wish to publish the results of this study, the investigator agrees to provide the sponsor with a manuscript for review 60 days prior to submission for publication. The sponsor retains the right to delete from the manuscript confidential information and to object to suggested publication and/or its timing (on the company’s sole discretion). | The present manuscript was drafted and finalised independently of the sponsor. The funding source had no role in study design, data analysis, data interpretation, or writing of the report. The corresponding author had full access to all the data in the study and had final responsibility for the decision to submit for publication. |
| An effort will be made to publish the results of this clinical trial on the basis of the integrated clinical trial report. The publication will be compiled by the Coordinating Investigator and the sponsor. Both parties should come to an agreement upon the submission of the manuscript. Publication by the investigator/co-investigators is only possible after the sponsor agreed. Prior publication of data is prohibited. | Employees of the sponsor contributed to the design of the study. The study was conducted by support from the contract research organization, [NAME OF THE ORGANIZATION]. The sponsor had no influence on the interpretation of the data or the writing of the manuscript. |
| The sponsor must have the opportunity to review all proposed abstracts, manuscripts, or presentations regarding this study 60 days prior to submission for publication/presentation. Any information identified by the sponsor as confidential must be deleted prior to submission, it being understood that the results of this study are not to be considered confidential. Sponsor review can be expedited to meet publication guidelines. | The authors who are not affiliated with the sponsor had access to all the study data, take responsibility for the accuracy of the analysis, and had authority over the preparation of the manuscript and the decisions about publication. |
| The local investigators agree not to publish individual patient data from their respective centres before the initial complete publication if not explicitly allowed by the chief investigator, statistician, and sponsor. The sponsor demands a copy of any manuscript or abstract before submission for publication within a reasonable time period (at least 20 working days for an abstract and 45 working days for a manuscript). By this the sponsor has the right to review the publication regarding correctness and to investigate whether confidential information is disclosed or additional information is necessary. | The sponsor participated in the development of the study design and provided the verum medication and funding, but did not participate in the collection, analysis, and interpretation of the data.  Final approval of manuscript: List of all academic authors – no industry employee on author list. |
| The results of this study will be published or presented at scientific meetings with the approval of the steering committee and in accordance with the main contract of the trial. In accord with standard editorial and ethical practice, the steering committee will generally support publication of multicentre trials only in their entirety and not as individual centre data. A coordinating investigator will be designated by mutual agreement to coordinate and prepare the final publication. There will be one main publication. All proposals or manuscripts of subsequent publications have to be submitted to the steering committee, reviewed and approved by this body. Since the sponsor is re-presented in the steering committee this allows the sponsor to protect appropriate to read information and to provide comments based on information from other studies that may not yet be available to investigators. | The sponsor had no access to the database or the interim analyses. The analyses were presented by the independent statisticians to the independent data monitoring committee without disclosure to the data center, the investigators, or the sponsor. The [TRIAL ACRONYM] steering committee was responsible for the decision to publish and for the content of the manuscript. The sponsor provided the drug and financial support. |
| To prevent premature disclosure or proprietary information and patentable inventions, and to project publication rights of the investigators in multicentre trials, the sponsor requests review of written or oral presentation before initial submission to any publisher or meeting proceedings, journals, meeting newspapers or organised poster sessions. | The study sponsor had no role in the design and conduct of the study, or in the collection, analysis, and interpretation of the data. [AUTHOR INITIALS] had access to all the data in the study, and took the final decision to submit for publication. |
| All publications, abstracts or presentations including data from the present trial will be submitted for review to the sponsor at least 15 working days for an abstract or presentation and 45 working days for a journal submission. The sponsor will review the communications for accuracy (thus avoiding potential discrepancies with submissions to health authorities), verify that confidential information is not being inadvertently divulged and provide any relevant supplementary information. | The sponsor of the study had no role in study design, data collection, data analysis, data interpretation, or writing of the report. The corresponding author had full access to all the data in the study and had final responsibility for the decision to submit for publication. |
| The sponsor acknowledges the investigators’ right to publish the entire results of the trial. Any such a scientific paper, presentation, communication or other information concerning the investigations described in this protocol must be submitted in writing to the sponsor trial manager prior to submission for publication presentation for comments. Comments will be given within four weeks from receipt of the manuscript. | The authors wrote the trial protocol and the manuscript, whereas the sponsor was responsible for collecting the data (data collection was performed by XXXX, a contract research organization). The authors had full access to the data, directed the data analysis, and were responsible for decisions regarding publication. The principal investigator [NAME OF ACADEMIC INVESTIGATOR] assumes full responsibility for the integrity and interpretation of the data. |
| The sponsor must have the opportunity to review all the proposed abstracts, manuscripts, or presentations regarding this study 60 days prior to submission for publication or presentation. Any information identified by the sponsor as confidential must be deleted prior to submission, it being understood that the results of the study are not to be considered confidential. The sponsor review can be expedited to meet publication guidelines. | Data were collected and analyzed initially by the sponsor. All data in the study were source-documented. All the authors had full access to the primary data and to their analysis. There was full independence in decisions concerning the reporting of results and the content of the manuscript, with no interference from the sponsor. |
