# Supplementary material for: Agreements between Industry and Academia on Publication Rights: A Retrospective Study of Protocols and Publications of Randomized Clinical Trials
Source: PLoS Med. 2016 Jun 28;13(6):e1002046. doi: 10.1371/journal.pmed.1002046 (PMC4924795; doi:10.1371/journal.pmed.1002046)
Supplement: S5 Table — Variables collected from protocols and publications for the entire cohort. (PDF) [file pmed.1002046.s006.pdf]

Variables 1 - 133 describe PROTOCOL specific characteristics.				
**#**	**form**	**variable**	**label**	**coding**
1	protocol	anchor_centerprot	Center and protocol information	section heading
2	protocol	center	Please indicate the name of the center and the ethics committee providing the protocol	text
3	protocol	archive_id	Enter local archive identification number of the protocol.	text
4	protocol	approval	Date of approval by ethics committee	date
5	protocol	studyachronym	Please provide the study acronym	text
6	protocol	studytitle	Please provide study title (quote!)	text
7	protocol	contact_appl	Contact data of the local applicant/local PI/investigator	section heading
8	protocol	title_appl	Title of the APPLICANT	Prof::Dr::Ms::Mr::unknown
9	protocol	familyname_appl	Family name of APPLICANT	text
10	protocol	gvname_appl	Given name of APPLICANT	text
11	protocol	institut_appl	Name of the department of the APPLICANT	text
12	protocol	street_appl	Street name and number of the APPLICANT	text
13	protocol	postcode_appl	Postal code APPLICANT	text
14	protocol	town_appl	Town of the APPLICANT	text
15	protocol	country_appl	Country of the APPLICANT	text
16	protocol	tel_appl	Telephon number APPLICANT	text
17	protocol	email_appl	Email of APPLICANT	text
18	protocol	contact_pi	Contact data of the overall principle investigator	section heading
19	protocol	contact_pipat	Are the contact data of the overall principle investigator given?	yes::no
20	protocol	piapplicant	Are the contact data of the local principle investigator and the overall principle investigator identical?	yes::no
21	protocol	title_pi	Title of the overall Principle Investigator	Prof::Dr::Ms::Mr::unknown
22	protocol	familyname_pi	Family name of the overall Principle Investigator	text
23	protocol	gvname_pi	Given name of the overall Principle Investigator	text
24	protocol	institut_pi	Name of the department of the overall Principle Investigator	text

#	form	variable	label	coding
25	protocol	street_pi	Street name and number of the overall Principle Investigator	text
26	protocol	postcode_pi	Postal code of the overall Principle Investigator	text
27	protocol	town_pi	Town of the overall Principle Investigator	text
28	protocol	country_pi	Country of the overall Principle Investigator	text
29	protocol	tel_pi	Telephon number of the overall Principle Investigator	text
30	protocol	email_pi	Email of the overall Principle Investigator	text
31	protocol	language	Language of the document	English::German::French::other
32	protocol	anchor_prop	Trial Properties	section heading
**33**	**protocol**	**pilot**	**Is the trial labeled as a pilot study?**	**yes::no**
**34**	**protocol**	**completed**	**Is the trial completed according to correspondence with ethics committee?**	**yes::no::unclear**
35	protocol	completed_yes	-if yes; please enter the exact date if given:	date
36	protocol	completed_no	- if no: specify reason (choose one)	stopped for benefit::stopped for harm:: stopped for futility::stopped for slow recruitment::other
**37**	**protocol**	**design**	**What is the trial design?**	**parallel::cross-over::factorial::other**
38	protocol	design_pararms	- if parallel design: indicate number of study arms	number
39	protocol	design_ratio	- if parallel design: indicate the ratio of randomization	number
40	protocol	multicenter	Are multiple centers included?	single center trial::multi center trial::unclear
41	protocol	multicenter_no	- if multi center: indicate the number of centers	number
42	protocol	multicenter_type	- if multi center: indicate type of multi-center	international::national study::unclear
**43**	**protocol**	**randomization**	**What is the unit of randomisation?**	**individuals::clusters::body parts**
**44**	**protocol**	**target_pop**	**What is the study population of the trial?**	**healthy individuals::patients suffering from disease or at risk for disease/event**
45	protocol	studypopulation	Please specify the study population.	text

#	form	variable	label	coding
46	**protocol**	**intervention**	**What is the type of experimental intervention (check all that apply)?**	**medication::surgery / invasive procedure::rehabilitation::behavioral intervention::diagnostic test::other**
47	protocol	intervention_desc	Describe experimental intervention(s) in detail.	description
48	**protocol**	**control**	**What is the type of control intervention (check all that apply)?**	**no active treatment / standard care::active (drug / other treatment)::placebo / sham procedure**
49	protocol	control_desc	Describe control intervention(s) in detail.	description
50	**protocol**	**studylabel**	**The study is a:**	**superiority trial::non-inferiority trial / equivalence::unclear**
51	protocol	anchor_funding	Study Funding	section heading
52	**protocol**	**funding**	**What is the funding source (check all that apply)?**	**government / public::charity / private not for profit::industry / private for profit::not funded / only in house source::not reported::reported, but type of funding unclear**
53	protocol	funding_industry	- if private for profit: what is funded?	medication/device only::more than medication/device::fully::unclear
54	protocol	funding_desc	Describe the funding source of the study.	funding description
55	**protocol**	**changes_prot**	**Does the correspondance/amendments between investigator and REB mention any important changes with respect to (check all that apply):**	**trial design (e.g. number of arms)::experimental/control intervention::sample size/enrollment issues::duration of follow up::primary outcomes/analysis::subgroups::none::other**
56	protocol	changes_text	Please specify any changes in the protocol:	text
57	protocol	publ_status_anchor	Status of Publication	section heading

#	form	variable	label	coding
58	protocol	publ_status	Has the trial of this protocol been published? (will be evaluated by the team in Basel, please see description before answering)	yes::no::not evaluated yet::unclear
59	protocol	publ_status_type	-if the trial was published, what was the type of publication?	publication in journal::only abstract for conference::only book::other
60	protocol	publ_status_typedescr	- if the trial was published, please specify the first author, title, name of e.g. journal, conference or book, and year.	text
61	protocol	publ_status_source	- if the trial was published, what is the source of this information?	protocol/correspondence with REB::literature search::author survey
62	protocol	publ_stop	Has the trial been stopped early? (MERGED information, please see description before answering)	yes::no::unclear::not evaluated yet
63	protocol	publ_stop_dep	-if trial was stopped early, what was the reason (check all that apply)?	stopped because of slow recruitment::stopped for benefit::stopped for harm::stopped for futility::other
64	protocol	publ_stop_report	-if trial was stopped early, what is the source of this information	protocol/correspondence with REB::publication::author survey
65	protocol	anchor_clinicalarea	Clinical Area and Setting	section heading
66	**protocol**	**clinicalarea**	**Clinical area is...**	**medical::surgical::paediatrics::other**
67	protocol	clinicalarea_med	- if medical area, choose from the list:	neurology::cardiovascular::respiratory::gastro/intestinal::nephrology::rheumatology::infectious disease::oncology::intensive care::hematology::endocrinology::dermatology::anaesthetics::psychiatry::other

#	form	variable	label	coding
68	protocol	clinicalarea_surg	- if surgical area, choose from the list:	general surgery::obstetrics / gynecology::neurosurgery::ophthalmology::ear-nose-throat (ENT)::cardiothoracic::urology::orthopedics::plastic surgery::other
69	protocol	clinicalarea_ped	- if paediatrics, choose from the list:	neurology::cardiovascular::respiratory::gastro/intestinal::nephrology::rheumatology::infectious disease::oncology::intensive care::hematology::endocrinology::dermatology::anaesthetics::general surgery::neurosurgery::ophthalmology::ear-nose-throat (ENT)::cardiothoracic::urology::orthopedics::plastic surgery::other
70	protocol	setting_pts	Setting and patient recruitment	section heading
71	**protocol**	**setting_spec**	**The setting for the majority of recruited patients is (check all that apply):**	**community::outpatient clinic::emergency department::in-patients hospital care::intensive care unit**
72	**protocol**	**setting_age**	**The age-group of patient population is (check all that apply):**	**adults (>=16 yrs)::only elderly (>=60 yrs)::pediatric(<18 yrs)**
73	protocol	anchor_outcomes	Outcomes	section heading
74	**protocol**	**primoutcome**	**Is any outcome specified as primary outcome?**	**yes::no**
75	protocol	primoutcome_prim	- if primary outcome is specified, only one or multiple outcomes?	only one outcome::multiple outcomes
76	protocol	primoutcome_txt	- if primary outcome is specified: please specify	primary outcome(s)
77	protocol	comendpnt	- if primary outcome is specified, is it a composite endpoint?	yes::no

#	form	variable	label	coding
78	protocol	comendpnt_specno	- if the primary enpoint is composite: specify number of components	number
79	protocol	outcometype	- if primary outcome is specified, provide types (check all that apply)	time to event::binary::continuous::count
80	protocol	patimp	-if primary outcome is specified, is it patient important?	yes::no
81	**protocol**	**qoloutcome**	**Quality of life specified as outcome?**	**yes::no**
82	protocol	outcomes_qolinstr	- if quality of life is specified as outcome: provide instrument used	text
83	**protocol**	**cost_eff**	**Any planned collection of costs or cost-effectiveness analysis mentioned?**	**yes::no**
84	protocol	anchor_analysis	Analysis	section heading
85	**protocol**	**specprimanalysis**	**Is a primary / main analysis specified?**	**yes::no**
86	protocol	specprimanalysis_adj	- if primary / main analysis is specified: is it...	adjusted for potential confounders::unadjusted for potential confounders
87	protocol	ittanalysis	- if primary analysis was specified, any intention to treat analysis (ITT) mentioned?	yes, ITT::yes, modified ITT::not reported
88	protocol	defitt	- if primary analysis was specified, any definition of intention to treat provided?	yes::no
89	protocol	ppanalysis	- if primary analysi was specified, any per protocol analysis mentioned?	yes::no

#	form	variable	label	coding
90	protocol	effectmeasure	- if primary analysis was specified, how were the results planned to be presented as (check all that apply)?	hazard ratio (HR)::relative risk or rate ratios (RR)::odds ratio (OR)::relative risk reduction (RRR)::risk difference/absolute risk reduction (ARR)::comparison of proportions/rates::comparison of Kaplan-Meier curves (log-rank test)::comparison of means / medians::not reported::other
91	protocol	lossfu	- if primary analysis was specified, any description of how investigators deal (will deal) with losses to follow up in the primary analysis (check all that apply)?	complete case analysis::assume nobody had the event::assume all had the event::worst case scenario::best case scenario::last observed value carried forward (LOCF)::censored (time to event analysis)::not reported::NO loss to follow up (for publication data extraction only)::other
92	**protocol**	**durationfu_prot**	**Please specify the planned duration of follow up of participants.**	**number**
93	protocol	anchor_subgroups	Subgroups	section heading
94	**protocol**	**plsubgrpanalysis**	**Any subgroup analysis mentioned?**	**yes::no**
95	protocol	plsubgrpanalysis_prespec	- if yes; is a clear hypothesis for a subgroup effect prespecified?	yes::no
96	protocol	plsubgrpanalysis_direffect	- if yes; is a clear hypothesis with direction of subgroup effect prespecified?	yes::no::not applicable
97	protocol	plsubgrpanalysis_interact	- if yes; use of interaction test for subgroup analysis mentioned?	yes::no
98	protocol	nosbgrvars_prot	- if yes, please specify number of subgroup analyses planned	number

#	form	variable	label	coding
99	protocol	anchor_samplerecr	Sample Size, recruitment, and safety	section heading
**100**	**protocol**	**totsampsze**	**Planned total sample size**	**number**
**101**	**protocol**	**samplszecalc**	**Sample size or power calculation mentioned?**	**yes::no**
102	protocol	totsampszeout	- if yes, what is the outcome used for sample size calculation?	text
103	protocol	checksmplszcalc	- if yes, check all provided elements of sample size calculation.	estimate / event rate in control group including measure of variability for continuous variable::treatment effect::type one error::power / type two error::assumption about losses to follow up
104	protocol	estevrateinctrgr	- if yes, what is the source of estimates for event rate/estimate in control group for sample size calculation (check all that apply)?	pilot study::review of patients in local institution::literature::not reported::other
105	protocol	smplpwr	- if yes, what power was assumed in sample size calculation (in %)?	number
106	protocol	estlossesfu	- if yes, estimated losses to follow up (%) in sample size calculation.	number
107	protocol	adjssmplsize	- if yes, any adjustments of sample size mentioned in amendments / correspondence during the trial?	yes::no
108	protocol	adjssmplsize_no	- if adjustments of sample size were mentioned: provide new target number	number
**109**	**protocol**	**interim**	**Any interim analysis?**	**yes::no**
110	protocol	interim_no	- if yes: specify the number	number
111	protocol	reasoninterim	- if yes, is the interim analysis for.. (check all that apply)	harm/safety::benefit/efficacy::futility::purpose not reported::other

#	form	variable	label	coding
112	protocol	definterim	- if yes, how was the interim period defined? (Check all that applies)	time(months)::no of recruited patients::no of events::after every event or patient::unclear::not reported::other count
113	protocol	monitor	- if yes, is any stopping rule / alpha spending function described?	yes::no
114	protocol	projection_recr	Projection of recruitment during planned enrollment time	section heading
115	**protocol**	**timerecr**	**Planned time period for patient recruitment**	**text**
116	**protocol**	**proj_recroverall**	**Is any patient recruitment projection mentioned?**	**yes::no**
117	protocol	proj_recroverall_facts	- If yes, the projection of recruitment is based on (check all that apply):	pilot or feasibility phase (INCLUDING informed consent and running FULL protocol)::pilot or feasibility phase (prospectively filling eligibility forms for patients WITHOUT formal consent)::retrospective check of patient volume with target disorder at ALL participating centers::retrospective check of patient volume with target disorder at one or some participating centers::estimated patient volume based on external information (e.g. literature, registry, national statistics)
118	protocol	meth_support	Presence of logistic/methodological support/experience	section heading

#	form	variable	label	coding
119	**protocol**	**trial_org**	**The protocol mentions (check all that apply):**	**a dedicated CENTRAL trial coordinator::dedicated paid LOCAL staff (recruitment/trial coordinater, clinical trial nurse) at participating centers::a dedicated trial coordinating/supporting unit/organization (e.g. CRO)::consumer/patient involvment in planing/conduct of trial::detailed description of professional data collection/management/monitoring::regular newsletters or progress reports to participants and/or trial staff::poster of information leaflets in clinics and wards::advertisement in newspapers::briefing of multidisciplinary teams (e.g. tumor boards) which can recommend patients for a specific trial::specific training held for staff recruiting patients::provision of training videos::regular visits/audits by PI/steering committee member or trial coordinator at recruitment sites::use of screening logs for all patients meeting inclusion criteria::none of these::other::none of these**

#	form	variable	label	coding
120	**protocol**	**money**	**Any financial incentive for patients or investigators mentioned?**	**yes::no**
121	protocol	pts_money	- if yes, for PATIENTS: Is there any financial incentive for participating patients (check all that apply) mentioned in the publication or protocol or in the patient consent form?	direct financial incentive (e.g. fixed amount of money)::remuneration for expenses (e.g. travel, parking etc to attend follow up visits)::none
122	protocol	invest_money	- if yes, for CENTERS/INVESTIGATORS: Is there any financial incentive for recruiting centers/investigators mentioned in the protocol? (please ONLY consider information from the protocol itself or patient consent form to answer this question)	fixed amount of money per patient::separate agreement on financial incentive mentioned::no financial incentive::not reported
123	**protocol**	**datasafetypubl**	**Does the study mention the presence of a data safety monitoring committee?**	**yes::no**
124	protocol	datasafetypubl_blind	- if yes: was (is) the committee blinded to treatment allocation of the groups?	yes::no::unclear
125	protocol	datasafetypubl_comp	- if yes: what is the composition of the board (check all that apply)	list of member names provided::member affiliations provided::member expertise provided::sponsor representative was member::not reported
126	protocol	publrules_head	Trial initiation and publication/stopping rules	section heading
127	**protocol**	**initiation**	**Is the trial industry or investigator initiated?**	**Definitely industry initiated::Probably industry initiated::Probably investigator initiated::Definitely investigator initiated**
128	**protocol**	**publrules**	**Does the protocol mention any rules/regulations between the investigators and the sponsor with respect to the rights of publication of the trial results?**	**yes::no::not applicable, because sponsor not from industry**

#	form	variable	label	coding
129	protocol	publrules_exp	- if yes, which statement suites best:	Only the sponsor retains the right to analyze and publish the data (no cooperation with investigators at all)::The sponsor retains the right to approve any manuscript/abstract before publication (sponsor retains explicitly the right to reject submission for publication)::The sponsor retains at least the right to review and comment on any manuscript/abstract before publication.::Free publication rights for the investigators, no constraints at all by the sponsor (sponsor has explicitly NOT the right to reject the submission for publication)::Protocol refers to a separate publication agreement between sponsor and investigator::Other
130	**protocol**	**stoptrial**	**Does the sponsor retain the right to stop the trial?**	**yes::no::not reported**
131	protocol	stoptrial_anyr	- if yes, explicitly at any time for any reason?	yes::no
132	protocol	stoptrial_desc	- if yes, for any specific reason (please specifiy)	text
133	protocol	comments	Any comments	text

		Variables 1 - 109 describe PUBLICATION specific characteristics		
#	form	variable	label	coding
1	publication	anchor_authorpubl	Author and publication information	section heading
2	publication	lastname	Last name of the first author	text
3	publication	journalname	Name of the Journal	text
4	publication	publicationyear	Year of publication	text
5	publication	pagespubl	First page or document number of journal	number
6	publication	registry	Does the publication mention a trial registration number?	text
7	publication	language	Language of the document	English::German::French::other
8	publication	anchor_trialprop	Trial Properties	section heading
9	publication	pilot	Is the trial labeled as a pilot study?	yes::no
10	publication	design	What is the trial design?	parallel::cross-over::factorial::other
11	publication	design_pararms	- if parallel design: indicate number of study arms	number
12	publication	design_ratio	- if parallel design: indicate the ratio of randomization	number
13	publication	design_cross_no	- if cross-over: indicate number of phases	number
14	publication	design_cross_wash	- if cross-over: washout between phases?	yes::no
15	publication	multicenter	Are multiple centers included?	single center trial::multi center trial::unclear
16	publication	multicenter_no	- if multi center: indicate the number of centers	number
17	publication	multicenter_type	- if multi center: indicate type of multi-center	international::national study::unclear
18	publication	randomization	What is the unit of randomisation?	individuals::clusters::body parts
19	publication	target_pop	What is the study population of the trial?	healthy individuals::patients suffering from disease or at risk for disease/event

#	form	variable	label	coding
20	publication	intervention	What is the type of experimental intervention (check all that apply)?	medication::surgery / invasive procedure::rehabilitation::behavioral intervention::diagnostic test::other
21	publication	control	What is the type of control intervention (check all that apply)?	no active treatment / standard care::active (drug / other treatment)::placebo / sham procedure
22	publication	studylabel	The study is a:	superiority trial::non-inferiority trial / equivalence::unclear
23	publication	anchor_funding	Study Funding	section heading
24	publication	funding	What is the funding source (check all that apply)?	government / public::charity / private not for profit::industry / private for profit::not funded / only in house source::not reported::reported, but type of funding unclear
25	publication	funding_industry	- if private for profit: what is funded?	medication/device only::more than medication/device::fully::unclear
26	publication	funding_desc	Describe the funding source of the study.	text
27	publication	authorindustry	Any investigator (author) or member of steering committee affiliated with industry?	yes::no::unclear
28	publication	authorindustry_unclear	- if industry affiliation 'unclear': specify	text
29	publication	authorindustry_yes	- if 'yes', specify the number of investigators with industry affiliation from total number of e.g. authors or members of the steering committee, whatever applies (e.g. 2/7):	number

#	form	variable	label	coding
30	publication	sponsorrole_publ	Does the publication explicitly mention the role of the sponsor in design and conduct of the trial and publication of the manuscript?	yes::no
31	publication	sponsorrole_publanswr	- if yes, which statement suites best with respect to publication rights:	Only the sponsor retained the right to analyze and publish the data (no cooperation with investigators at all)::The sponsor retained the right to approve the manuscript/abstract before publication (sponsor retains explicitly the right to reject submission for publication)::The sponsor retained at least the right to review and comment on the the manuscript/abstract before publication.::Free publication rights for the investigators, no constraints at all by the sponsor (sponsor had explicitly NOT the right to reject the submission for publication)::Other
32	publication	anchor_clinicalarea	Clinical Area and Setting	section heading
33	publication	clinicalarea	Clinical area is...	medical::surgical::paediatrics::othe r

#	form	variable	label	coding
34	publication	clinicalarea_med	- if medical area, choose from the list:	neurology::cardiovascular::respiratory::gastro/intestinal::nephrology::rheumatology::infectious disease::oncology::intensive care::hematology::endocrinology::dermatology::anaesthetics::psychiatry::other
35	publication	clinicalarea_surg	- if surgical area, choose from the list:	general surgery::obstetrics / gynecology::neurosurgery::ophthalmology::ear-nose-throat (ENT)::cardiothoracic::urology::orthopedics::plastic surgery::other
36	publication	clinicalarea_ped	- if paediatrics, choose from the list:	neurology::cardiovascular::respiratory::gastro/intestinal::nephrology::rheumatology::infectious disease::oncology::intensive care::hematology::endocrinology::dermatology::anaesthetics::general surgery::neurosurgery::ophthalmology::ear-nose-throat (ENT)::cardiothoracic::urology::orthopedics::plastic surgery::other
37	publication	anchor_methodsquality	Methodological Quality	section heading

#	form	variable	label	coding
38	publication	randoconceal	Randomization and concealment (check all that apply)	central randomization::sealed, opaque, and sequentially numbered envelopes::envelopes, other::open random allocation schedule::quasi-randomized::probably concealed, but not explicitly described::not concealed::not reported
39	publication	blindingpts	Blinding of patients/participants	definitely yes::probably yes::probably no::definitely no
40	publication	blindinghealthcare	Blinding of health care providers	definitely yes::probably yes::probably no::definitely no
41	publication	blindingdatacoll	Blinding of data collectors	definitely yes::probably yes::probably no::definitely no
42	publication	blindingoutcadj	Blinding of outcome adjudicators	definitely yes::probably yes::probably no::definitely no
43	publication	blindinganalysis	Blinding of data analysts	definitely yes::probably yes::probably no::definitely no
44	publication	stoppingearly	Was the trial stopped early?	no::yes, for benefit::yes, for harm::yes, for futility::yes, slow recruitment::yes, other reason
45	publication	anchor_enrolment	Enrolment and Follow Up	section heading
46	publication	startingenrol	Starting date of enrolment (dd.mm.yyyy)	date
47	publication	closingenrol	Closing date of enrolment (dd.mm.yyyy)	date
48	publication	duration	Duration of enrolment in months	number
49	publication	closingfu	Closing date of follow up (dd.mm.yyyy)	date
50	publication	durationfu	Duration of follow up of participants reported?	yes::no

#	form	variable	label	coding
51	publication	typefumeasure	-if follow up duration is reported, please specify the type of measure (check all that apply):	fixed period::mean::median::minimum::maximum::not reported
52	publication	unitoffutime	-if follow up duration is given, provide the unit	days::weeks::months::years
53	publication	fupoint	-if follow up duration is given, specify the value:	number
54	publication	anchor_outcomes	Outcomes	section heading
55	publication	primoutcome	Is any outcome specified as primary outcome?	yes::no
56	publication	primoutcome_prim	- if primary outcome is specified, only one or multiple outcomes?	only one outcome::multiple outcomes
57	publication	primoutcome_txt	- if primary outcome is specified: please specify	text
58	publication	comendpnt	- if primary outcome is specified, is it a composite endpoint?	yes::no
59	publication	comendpnt_specno	- if the primary enpoint is composite: specify number of components	number
60	publication	outcometype	- if primary outcome is specified, provide types (check all that apply)	time to event::binary::continuous::count
61	publication	patimp	-if primary outcome is specified, is it patient important?	yes::no
62	publication	outcomesign	- if primary outcome is specified, is any of the primary outcomes statistically significant?	significant::not significant
63	publication	qoloutcome	Quality of life specified as outcome?	yes::no
64	publication	outcomes_qolinstr	- if quality of life is specified as outcome: provide instrument used	text
65	publication	anchor_analysis	Analysis	section heading
66	publication	noofptsapproached	Is the number of patients assessed for eligibility reported?	yes::no
67	publication	noofptsapproached_nr	- if yes: specify number	number
68	publication	ptsrando	Total number of patients randomized to all arms	number
69	publication	postrandoex	Post randomization exclusions?	yes::no

#	form	variable	label	coding
70	publication	postrandoex_number	- if post randomization exclusions are reported: specify number	number
71	publication	checkfu	Is lost to follow up (LTFU) explicitly reported?	yes::no
72	publication	ptsrandofu	Number of patients with missing outcome data from all arms (lost to follow up)	number
73	publication	specprimanalysis	Is a primary / main analysis specified?	yes::no
74	publication	specprimanalysis_adj	- if primary / main analysis is specified: is it...	adjusted for potential confounders::unadjusted for potential confounders
75	publication	effectmeasure	The results of the primary analysis are presented as (check all that apply)?	hazard ratio (HR)::relative risk or rate ratios (RR)::odds ratio (OR)::relative risk reduction (RRR)::risk difference/absolute risk reduction (ARR)::comparison of proportions/rates::comparison of Kaplan-Meier curves (log-rank test)::comparison of means / medians::not reported::other
76	publication	clustertrial_method	- if yes, which method was used (check all that apply)	Mantel-Haenzel method::random effects model / hierarchical regression / generalized linear mixed model::not reported::not applicable
77	publication	ittanalysis	Any intention to treat analysis (ITT) mentioned?	yes, ITT::yes, modified ITT::not reported
78	publication	defitt	Any definition of intention to treat provided?	yes::no
79	publication	ppanalysis	Any per protocol analysis mentioned?	yes::no

#	form	variable	label	coding
80	publication	lossfu	Any description of how investigators deal (will deal) with losses to follow up in the primary analysis (check all that apply)?	complete case analysis::assume nobody had the event::assume all had the event::worst case scenario::best case scenario::last observed value carried forward (LOCF)::censored (time to event analysis)::not reported::NO loss to follow up (for publication data extraction only)::other
81	publication	anchor_subgroups	Subgroups	section heading
82	publication	plsubgrpanalysis	Any subgroup analysis mentioned?	yes::no
83	publication	sg_pres	- if yes, do the authors report that the subgroup analyses were pre specified?	yes::no
84	publication	sg_adhoc	- if yes, do the authors report that the subgroup analyses were done post hoc?	yes::no
85	publication	sg_preadhoc	- if yes, do the authors report that the subgroup analyses were pre specified AND and ad hoc?	yes::no
86	publication	sg_pwr	- if yes, do the authors report any power calculation for subgroup analyses?	yes::no
87	publication	plsubgrpanalysis_prespec	- if yes; is a clear hypothesis for a subgroup effect prespecified?	yes::no
88	publication	plsubgrpanalysis_direffect	- if yes; is a clear hypothesis with direction of subgroup effect prespecified?	yes::no::not applicable
89	publication	plsubgrpanalysis_interact	- if yes; use of interaction test for subgroup analysis mentioned?	yes::no
90	publication	nosbgrvars	- if yes, please specify number of subgroup analyses reported	number
91	publication	subgroupanalclaim	Any claims of subgroup effects in publication?	yes::no

#	form	variable	label	coding
92	publication	anchor_samplerecr	Sample Size, recruitment, and safety	section heading
93	publication	totsampsze	Planned total sample size	number
94	publication	samplszecalc	Sample size or power calculation mentioned?	yes::no
95	publication	totsampszeout	- if yes, what is the outcome used for sample size calculation?	text
96	publication	checksmplszcalc	- if yes, check all provided elements of sample size calculation.	estimate / event rate in control group including measure of variability for continuous variable::treatment effect::type one error::power / type two error::assumption about losses to follow up
97	publication	estevrateinctrgr	- if yes, what is the source of estimates for event rate/estimate in control group for sample size calculation (check all that apply)?	pilot study::review of patients in local institution::literature::not reported::other
98	publication	smplpwr	- if yes, what power was assumed in sample size calculation (in %)?	number
99	publication	estlossesfu	- if yes, estimated losses to follow up (%) in sample size calculation.	number
100	publication	changesmplsze	Changes in sample size during trial reported?	yes::no
101	publication	changesmplsze_yes	- if yes, please provide the new sample size.	number
102	publication	interim	Any interim analysis?	yes::no
103	publication	interim_no	- if yes: specify the number	number
104	publication	reasoninterim	- if yes, is the interim analysis for.. (check all that apply)	harm/safety::benefit/efficacy::futility::purpose not reported::other
105	publication	monitor	Is any stopping rule / alpha spending function described?	yes::no
106	publication	datasafetypubl	Does the study mention the presence of a data safety monitoring committee?	yes::no

#	form	variable	label	coding
107	publication	datasafetypubl_blind	- if yes: was (is) the committee blinded to treatment allocation of the groups?	yes::no::unclear
108	publication	datasafetypubl_comp	- if yes: what is the composition of the board (check all that apply)	list of member names provided::member affiliations provided::member expertise provided::sponsor representative was member::not reported
109	publication	comments	Any comments	text
